# Oral health in asthmatic patients: a review: Asthma and its therapy may impact on oral health

**DOI:** 10.1186/s12948-020-00137-2

**Published:** 2020-11-07

**Authors:** Federica Gani, Marco Caminati, Fabio Bellavia, Andrea Baroso, Paolo Faccioni, Paolo Pancera, Veronica Batani, Gianenrico Senna

**Affiliations:** 1 Allergy Service AOU San Luigi Gonzaga Orbassano, Turin, Italy; 2 Department of Medicine, University of Verona, Piazzale Scuro, Verona, Italy; 3 Allergy Unit and Asthma Center, Verona University Hospital, Piazzale Scuro, Verona, Italy; 4 Section of Oral and Maxillofacial Surgery, Department of Surgical Sciences, Dentistry, Gynecology and Pediatrics, University of Verona, Verona, Italy

**Keywords:** Asthma, Corticosteroid inhalers, Dental caries, Oral candidiasis, Periodontal disease, Saliva

## Abstract

Different drugs used to treat asthma, such as beta 2 agonists and inhaled steroids, may promote a higher risk of caries, dental erosion, periodontal disease and oral candidiasis. This article reviews the evidences of mechanisms involved in oral diseases in patients affected by asthma. The main mechanism involved is the reduction of salivary flow. Other mechanisms include: acid pH in oral cavity induced by inhaled drugs (particularly dry powder inhaled), lifestyle (bad oral hygiene and higher consumption of sweet and acidic drinks), gastroesophageal reflux, and the impairment of local immunity. In conclusion asthma is involved in the genesis of oral pathologies both directly and indirectly due to the effect of the drugs used to treat them. Other cofactors such as poor oral hygiene increase the risk of developing oral diseases in these patients. Preventive oral measures, therefore, should be part of a global care for patients suffering from asthma.

## Introduction

Asthma is a chronic inflammatory disease characterized by increased airway responsiveness to various environmental triggers and by symptoms such as wheezing, coughing, chest tightness and dyspnoea. The diagnosis is based on clinical evaluation and functional demonstration of reversible airway obstruction, either spontaneously or after medical treatment [1]. Different mechanisms of inflammation are involved in the pathogenesis, leading to heterogeneous phenotypes of the disease. This includes T Helper 2 (TH2) pathway in which eosinophils play the main role (this group includes allergic but also non allergic asthma), and the non TH2 pathway with neutrophilic, mixed or paucicellular infiltrates [1].

Asthma is a growing health problem affecting over 300 million people worldwide. The recent Global Strategy for Asthma Management and Prevention Report shows that 15% of Italian children suffer from wheezing and 11.6% from lifetime asthma. For adults, these percentages decrease to 6% and 10% respectively and women result more affected by wheezing than men: 6.64% versus 5.49%. Women also have a peak prevalence between the ages of 45 and 55 years old. Otherwise, the disease tends to decrease in old age [1].

The aim of asthma treatment is to reduce airways inflammation and to induce bronchodilation in order to control symptoms and prevent the progression of the disease and the onset of exacerbations [1]. Treatment is chosen based on the severity of the disease. The choices include mainly short acting bronchodilators (these are not used alone but only in combination with anti-inflammatory drugs), anti-inflammatory agents such as CSI (or oral CS used only in selected severe patients), long acting bronchodilators, and leukotriene modifiers. Only severe forms of asthma should be treated with biologic drugs [1].

Many inhaled drugs used to treat asthma may have systemic effects.

*Inhaled corticosteroids* systemic effects impact on the hypothalamo-pituitary-adrenal (HPA) axis, as well as on bones, skin, eyes, growth and immunity. Systemic effects are dose-related and their relevance depends on patient’s susceptibility regardless of steroid type. However different molecules are associated with differently relevant side effects, being budesonide responsible for a few systemic effects whilst fluticasone having a higher risk especially at doses above 400 mcg/day [2]. In asthma many studies have shown that ICS have an excellent safety profile at the low doses usually required, since adverse effects appear mostly at higher doses [3].

*Inhaled beta2*-*agonists* at high dose also cause dose-related systemic adverse effects including tremor, tachycardia, hypokalaemia and are associated to electrocardiographic sequelae, which are mediated by extrapulmonary receptors. Systemic absorption occurs primarily across the lung-vascular bed and hence compounds which are more lipophilic, such as fenoterol, are absorbed to a greater extent [4].

*Inhaled anticholinergics* (ex. tiotropium) can cause side systemic effects that include cardiac events such as tachycardia and gastrointestinal involvement such as constipation, as well as urinary retention and urinary tract infections. Concerning tiotropium (the only anticholinergic drug approved in severe asthma) it has been reported in asthmatic adults and in children that there were low or absent systemic side effects in all age groups [5].

At the same time, these drugs can have local effects being involved in oral disease through different pathways, promoting a higher risk of caries, dental erosion, tooth loss, periodontal disease and oral candidiasis [6]. Furthermore, asthmatic patients, independently from taking drugs, may show also a modification in stimulated salivary components -such as a decrease in total protein, amylase, hexosamine, salivary peroxidase, lysozyme and secretory IgA—that further impairs drug induced xerostomia [6]. Moreover there are evidences that bronchial asthma itself can be considered a risk factor for increased gingival inflammation and dental erosion and that its severity is directly related with a higher risk for dental caries and gingival affection [7]. The aim of this review is to analyse the mechanisms involved in the developing of oral lesions and pathologies in asthmatic patients.

## Asthma and oral health

### Asthma and the oral environment: effects on salivary flow and oral mucosa

Saliva is composed of water and a range of inorganic and organic components, which allow oral protection from micro-organisms, lubrication for mastication, and protection for tooth structures [8].

The physiological functions of the saliva are to supply nourishment to the oral microbiota, to carry antimicrobial factors, and to contribute to the maintenance of microbial homeostasis [9].

Over 700 different bacterial species have been identified in the oral microbiota [10, 11]. Firmicutes are the most frequent bacteria phylum found in the human saliva [12].

Salivary flow and composition play a fundamental role in oral health, with a defensive mechanism that can be impaired in the asthmatic condition due to medications used and alimentary habits or lifestyles [7].

Saliva is involved in maintaining a neutral pH in the oral cavity sometimes reaching values up to 7.67 depending on different types of buffering systems such as bicarbonate, phosphate, urea, and amino peptides [8]. Salivary flow allows a mechanical cleansing against food debris or microbial agents, and oral clearance is related to the rate of secretion [6]. These conditions are impaired in asthma as beta 2 agonists revealed a negative effect on the salivary production rate and many inhalers have a low pH [7].

Three studies conducted between 1991 and 1998 about the effect of beta 2 short acting agonists, taken over a period of 7 days, reported no reduction in salivary flow. A study of 15 patients with moderate persistent asthma treated with salmeterol 50 mcg plus fluticasone propionate 100 mcg twice daily, for 1 month, demonstrated a reduction in salivary flow rate associated to an increase in dental plaque index and no modification in salivary IgA [13]. In another study conducted in 2001, 30 patients with moderate to severe asthma were treated for 6 weeks with salmeterol alone or in combination with beclomethasone. It demonstrated that both treatments induced injuries in mouth mucosa without modification of salivary flow, indicating that it was not the only protective factor for gingivitis [14].

Recently, tiotropium (a long-acting anticholinergic inhaled drug-LABA) has been approved as an add-on controller medication in patients with severe asthma [15]. Xerostomia is a typical side effect of the anticholinergic therapeutic class, and it has been investigated in a pooled safety analysis of 3474 adults with symptomatic asthma from seven phase II and III clinical trials. Dry mouth was reported by 1% of patients in the tiotropium 5 µg group and 0.5% of patients in the placebo group. Similar results were found for the tiotropium 2.5 µg pooled group versus placebo (0.4% and 0.5% of patients, respectively). Finally dry mouth was not considered relevant and did not lead to treatment discontinuation in any patients [16]. In adolescents and children the Cano-TinA-asthma^®^ trial reported that there were no evidence of dry mouth in patients treated with LABA [17]. In conclusion, dry mouth was low or absent in all age groups treated with tiotropium that therefore is an additional well-tolerated and effective therapeutic option for the treatment of severe poorly controlled severe asthma [5].

The reduced salivary flow could result in lower antimicrobial protection and oral dehydration of the mucosa so that, in order to prevent xerostomia, these patients intake a large amount of products that could be cariogenic. In fact, an increasing consumption of drinks that have low pH was noted and could be involved in erosive lesions. Lifestyle choices of asthmatic children, with frequent consumption of sweets, could also promote oral lesions [7].

Furthermore, the inhaled therapy is often taken at night before going to bed without any oral hygiene and the lack of masticatory movements might increase the damage in the oral environment due to the medications themselves [7].

## Asthma and candidiasis

Inhaled steroids play a central role in asthma treatment due to their anti-inflammatory effect [1] and their reduction of asthma mortality.

Oropharyngeal candidiasis is common in people using nebulized corticosteroids [6], with a seemingly direct correlation between high doses and the time of exposure. Oral candidiasis typically includes symptoms such as an unpleasant feeling in throat and pharyngodynia. This discomfort can lead to discontinuation of the therapy [18]. Oral candidiasis is mainly caused by the immunosuppressive effects of ICS [19] and a decrease in salivary IgA and histatin [18]. Evidence has shown that only about 20% of the inhaled dose actually reaches the lungs, with the major amount remaining in the oropharynx. Furthermore, many of the dry powders inhalers (DPI) contain lactose monohydrate as a carrier which may worsen this disease. Beta 2 agonists may also contribute to a higher incidence of candidiasis by reducing the salivatory rate [6] Table 1.

**Table 1:** Possible causes of oral candidiasis

Lactose contained in the dry powder of inhalers that could promote candida’s growth and proliferation	
Low salivary flow rate due to beta 2 agonists	
Immunosuppressive and anti-inflammatory effects of steroids	
Decreased levels of IgA	

A study conducted in Japan in 2003 enrolled 143 asthmatics under treatment with inhaled steroids (96 with fluticasone propionate Diskhaler at 50, 100, or 200 μg per dose and the remaining 47 with beclomethasone dipropionate 100 μg per dose), 11 asthmatics patients not treated with CSI and 86 healthy controls. The data confirmed that a larger amount of Candida spp. organism was detected in asthmatic patients treated with CSI (p < 0.001). The aim of the study was also to evaluate the incidence of oral candidiasis in patients treated with fluticasone propionate through a dry powder inhaler rather than in patients treated with beclomethasone dipropionate administered using a pressurized metered-dose inhaler with a spacer. Candida was detected in 26% of asthmatics treated with fluticasone and in 10% of those treated with beclomethasone (p = 0.02). The inhaled dose of fluticasone was approximately 50% of beclomethasone and both the detection rate and the amount of candida were significantly higher in patients treated with fluticasone. It was concluded that beclomethasone use did not correlate with the amount of candida whereas there was a direct correlation between the amount of candida and the dose of fluticasone. The explanation could be that fluticasone, despite the major anti-inflammatory effects which requires low doses for achieving the therapeutic effect, may impair local immunity due to major solubility, strong inhibition of T cell migration and proliferation, and impaired viability of IL 5 induced eosinophils activation. In addition, the flow rate seems to impact on oral candidiasis susceptibility [18].

These findings were confirmed in a later study that evaluate the use of Fluticasone Propionate (FP) as a dry powder inhaler. In this study, four groups of patients were enrolled: 62 asthmatic patients who were taking 200 μg/day of FP, 122 asthmatics who were taking 500 μg/day of FP, 50 asthmatic patients who had not been on inhaled corticosteroid (ICS) treatment and 40 non-asthmatic subjects. The frequency of positive swabs for Candida colonization was higher in the 500 μg/day FP group than in the asthmatics without ICS (p < 0.05) and it was also higher than normal controls (p < 0.05), whereas there weren’t differences between the 200 μg/day FP group and controls. The most influential variables for Candida colonization in patients who used ICS were the washing of patients’ throats (p < 0.0001) and the duration of ICS treatment more than 12 months (p < 0.05) [20].

In 2007 a study was conducted to evaluate the risk of oropharyngeal and esophageal candidiasis in 40 asthmatic patients above the age of 18 compared to controls. The patients were treated with inhaled steroids (400 mcg to 1600 mcg of budesonide or fluticasone) for at least 1 month. The study distinguished between infection (clinical symptoms plus positive cultures) and colonization (only positive cultures). The results showed oesophageal candidiasis and oropharyngeal candidiasis in 2.5% and 5% of asthmatic patients respectively, while no differences were detected in candida colonizations in the two sites between the asthmatic group and the control group. These findings are not yet conclusive [21].

In order to minimize the incidence of oral candidiasis many preventive measures can be adopted, such as rinsing the mouth and using a spacer device, administering topical antimycotics (e.g. nystatin), using sialogogue medications in patients with low salivary flow rate, and chewing sugar free gums [6]. Moreover gargling a solution of amphotericin diluted 1:50 resulted in reducing the amount of candida and improving symptoms rather than just gargling water [18].

In summary, the main causes of oral candidiasis could be inhaled steroids (type and dose), This could be prevented by a correct mouth rinsing and using the lowest ICS dose to control the disease.

## Asthma and dental erosions

Dental erosion is characterized by the loss of enamel and dentin due to acid action. The pH threshold for dental enamel demineralization is below 5.5 [8]. The prevalence in the general population is 30.4% as documented by a systematic review conducted in 2015 [22].

Asthma medications can lead to a higher risk of dental erosion by reducing salivary protection against extrinsic (such as soft drinks, fruit juices, and dietary supplements,) and intrinsic acids (such as gastric acids in Gastroesophageal reflux disease-GERD) [6–8].

Furthermore, the main asthmatic drugs, especially the powdered ones, create a pH of less than 5.5, which is the critical level for hydroxyapatite dissolution [8] (Table 2).

**Table 2:** Possible causes of dental erosion

Reduction of salivary flow rate due to beta 2 agonists	
Increase in teeth’s exposure to acid	
Extrinsic sources: acid soft drinks, acidity of medications (inhalers, in particular dry powder)	
Intrinsic sources: gastroesophageal reflux	

Conclusions about the association between dental erosion and asthma have been controversial. A British study conducted in 2000 aimed to assess the level of dental erosion in a random sample of 418 subjects (14 year-olds), 15.8% of them asthmatics. It concluded that the level of dental erosion was higher in asthmatic children than in healthy controls (30% scored 2 vs 24% scored 2 using Tooth Wear Index.) [23].

Another study conducted later in the UK (2003) including more than 1300 (12 years-olds) asthmatic and non-asthmatic children, who were re-examined 2 years later, did not demonstrate a significant difference in erosion prevalence between the two groups. These results can be due to the fact that despite 88% of patients in the asthmatic cohort having drugs prescribed to them, they showed a pH above the critical level of 5.5 [24]. These data underline the importance of other cofactors besides drug therapy in the development of dental erosion. In fact in 1998 O’ Sallivan found various other risk factors that are associated with dental erosion which include: oral dryness due to the effects of bronchodilators and mouth breathing, consumption of drinks with low pH, and high acidity and gastroesophageal reflux (GOR) [25].

The relationship between gastroesophageal reflux and dental erosion has been proven [26] and the association between asthma and GERD is now well documented [1]. A cross sectional study, conducted in 2017 in a sample of 1869 subjects (18 year-olds), detected erosion in 42.3% of them via a clinical assessment using the Basic Erosive Wear Examination (BEWE). Among the comorbidities investigated, asthma, allergies, eating disorders and GOR were found to be 2.7%, 8.5%, 1.4% and 1.3% respectively. The statistical analysis revealed that the prevalence of erosion in the anterior region was related to gastroesophageal reflux, eating disorders, and asthma with a significance level of p < 0.05 [27].

GOR symptoms are more prevalent in asthmatic patients (approximately 75%) compared to controls [28] and the mean prevalence of dental erosion in adult patients affected by GOR is 32.5% [29]. It is assumed that antiasthmatic drugs delivered with DPI (which show lower pH compared to when administered with MDI), are associated with more dental erosion. There is no clear difference, however, in the degree of tooth erosion noticed using the two different way of drug administration. This could demonstrate the importance of GOR and other concomitant factors including lifestyle and oral hygiene in determining dental erosion [30].

A recent cross sectional study (2019) involving 400 children aged 6 to 14 years old, evaluated the relationship between dental erosion and etiological factors. The patients’ risk level of developing dental erosion was obtained from the BEWE index. A statistically significant relationship (p = 0.05) between the intake of carbonated beverages, isotonic drinks and fruit juices and a higher BEWE was detected. Furthermore, regarding the use of drugs that could be potentially erosive, a statistically significant correlation (p = 0.006) was shown with the BEWE index in patients using inhalers (5.3% of the total) [30].

Interventions such as rinsing the mouth with sodium bicarbonate or using neutral sodium fluoride mouth rinses after using inhalers [6], using spacer devices, and treating GOR symptoms, could be helpful in treating and preventing dental erosion [25].

In summary, dental erosion may be associated with the use of beta 2 agonists, independently from the type of inhaler, but cofactors like lifestyle, associated drugs, or GERD can increase the risk. Thus, rinsing the mouth and controlling cofactors is mandatory.

## Asthma and periodontal disease (gingivitis and dentin sensitivity)

The periodontium is composed of gingivae, cementum, the alveolar bone, and the periodontal ligament. The initial form of periodontal disease is inflammation of the gingivae (gingivitis) which become swollen, shiny, and easily bleeding. Periodontal health seems to be strongly connected to a proper salivary flow [7]. Periodontitis with severe attachment loss on multiple teeth is prevalent among approximately 0.2–0.5% of children and young adults [31].

Some studies report a significant amount of accumulated plaque and gingival inflammation in asthmatics [32]. Early in 1998, a study conducted in 100 asthmatic children revealed that the asthmatic population had significantly more plaque, gingivitis and calculus and more tooth surface loss compared to healthy controls [33].

A study was conducted in twenty older asthmatic patients (18–24 years old) with a mean duration of asthma of 13.5 years and twenty matched healthy controls. It demonstrated that the frequency in gingival inflammation was higher (p = 0.01) and the salivary flow was lower (p = 0.01) in the asthmatic group [34].

In addition, a study published in 2017 showed that gingival inflammation (considering the Gingival Index-GI) is more prevalent in asthmatic children than in corresponding controls and that it increases with the severity of asthma [7].

A systematic review conducted in 2018, including 21 studies published between 1979 and 2017, showed a strong association between asthma and periodontal disease [35].

Another study published 1 year later, including a systematic review of 11 studies and meta-analysis analyzing six parameters (plaque index, gingival index, bleeding on probing, papillary bleeding index, calculus index, clinical attachment loss,) confirmed a possible association of asthma with periodontal diseases in adults, especially regarding gingivitis (compared to healthy individuals). However, the authors outlined that further studies with similar methods are required to evaluate the interaction between these diseases [36]. A cross sectional comparative study conducted in 2016 among 40 asthmatic patients (18–60 years old) showed a higher rate of dentin sensitivity (35.13%) compared to controls (14.3%) (p = 0.00). These results, however, were not confirmed in another study where no difference was found between the two groups. This was probably due to differences between using toothpastes with desensitizing agents or not and other types of tooth wear with protective effects against dentin hypersensitivity [30].

The periodontal involvement in asthma could be due to immune and inflammatory processes, to side effects of asthma drugs, or to both [6]. The main cause of impairment of periodontal tissue is probably the reduced protective effect of saliva during oral dryness. This can be caused by mouth breathing [6], the alteration of salivary composition, or the reduction of salivary flow [7]. This phenomenon can promote the interaction between bacterial and immunological factors, including lower concentration of salivary IgA [6]. An increased concentration in IgE in the gingival tissues and a higher incidence of calcium and phosphorous with higher prevalence of calculus in the saliva, can also be involved in poor periodontal health [6].

Furthermore, ICS may reduce bone mineral density, including in the mandible [37]. Long term use of ICS in adults can lead to an increase in fractures, particularly in those who receive moderate to high doses. This can occur even if the systemic drug bioavailability is minimal [38]. For these reasons, ICS may have an impact on the onset and progression of periodontal disease and mandibular bone mineral density should be checked regularly if other risk factors for osteoporosis are associated. Therefore, disease control using the lowest ICS dose is one of the most important practices in asthma therapy [1] Table 3.

**Table 3:** Possible causes of periodontal disease

Dehydration of alveolar mucosa due to mouth breathing	
Alteration of immune response with an increased concentration of IgE and a reduction of secretory IgA	
Reduction in salivary flow due to beta 2 agonists	
Higher incidence of calculus due to an increased level of calcium and phosphorous in saliva	
Decrease in bone mineral density due to inhaled steroids	

A retrospective study involving a sample of 19,206 asthmatics, found that the hazard of developing periodontal disease increased sharply for patients with a more severe form of asthma (multiple emergency department visits or hospital admission). An association between ICS treatment and a higher risk of periodontal disease was detected [39]. However, this study contained several limitations in correctly defining asthma and periodontitis: a lack of information about the severity of the disease, the dose of cumulative steroids inhaled, and the short follow up period (about 6 years while periodontal disease develops slowly).

Not only asthma, but also allergic rhinitis and other respiratory diseases promoting mouth breathing could play an important role in developing periodontal affection. For example, obstructive sleep apnea (OSA), adenoid and tonsillar hypertrophy, neuromuscular disorders can affect young children [7].

## Asthma and dental caries/decay

Dental caries are one of the most common chronic diseases in children and they have been proven to affect systemic health and nutritional status, with a prevalence from 60 to 90% in school children [40]. Its pathogenesis is multifactorial, comprehensive of environmental, behavioral, and genetic factors, and involves a complex process of enamel dissolution and re-mineralization by organic acids produced by microorganisms within dental plaque [8].

Several studies have demonstrated a strong association between asthma and dental caries, particularly in children, although some other studies were not able to prove this relationship. A possible explanation of the differences between studies could be explained by different fluoride exposures, medication uses, and delivery devices (dry powder, spacers) [8].

Studies also suggest that prevalence of caries increases with the severity of asthma and the duration of treatment [6]. As early as 1987, some authors did not confirm these findings, [41] and in 2004 a study conducted in 140 asthmatic patients (7 to 17 years old) revealed that neither the period of the disease nor the use of medications nor the severity of asthma had a significant influence on the risk of caries and gingivitis. Oral hygiene (79% of children reported a proper oral hygiene) and dietary habits could not explain this lack of correlation [42].

On the other hand, a systematic review and meta-analysis conducted in 2010 evaluated the effects of asthma on primary dentition (11 articles) and permanent dentition (14 articles) and suggested that asthma doubles risk of caries in both primary and permanent dentition. Although there may be an overestimation of the odds ratio for permanent dentition but not for the primary one found [43].

Another study conducted in an older population (twenty 18–24 years old patients) with a long term duration of asthma (13.5 years), demonstrated a higher incidence of initial caries (6 vs 1.3) and lower salivary secretion rate than in controls without asthma [34].

More recent data come from a study conducted in Korea in 2018. A sample of 44,203 subjects including 1264 patients with asthma, were divided into groups based on the age of diagnosis of asthma (0 to 64 years). Asthma diagnosis in patients younger than 12 years showed a significant correlation with tooth loss due to caries rather than subjects with a delayed diagnosis [44]. Different mechanisms are involved in the increase of caries prevalence in asthmatic patients (Table 4).

**Table 4:** Possible causes of dental caries

Decrease of salivary flow rate induced by beta 2 agonist	
Increase in Lactobacilli and Streptococcus mutant bacteria in saliva	
Decrease in the salivary pH due to the use of inhalers	
Fermentable carbohydrate present in antiasthma medications	
Increased consumption of sweet food and drinks	

Ryberg et al. found that the risk of caries in asthmatic children seemed to be associated with the use of beta 2 agonists and consequently lower salivary flow increasing the amount of Lactobacilli and *Streptococcus mutans*. In addition, inhaled beta 2 drugs are connected with a significant decrease in salivary and plaque pH [6]. Some dry powdered drugs contain sugar (as lactose monohydrate), that are associated with reduced salivary flow, may contribute in developing of caries [6]. Also, as mentioned before, an increase in consumption of cariogenic foods (such as sweet drinks, or sweet) in the asthmatic population, is common.

Milano et al. conducted a study in 179 asthmatic children to examine the relationship between the types of drugs, the frequencies of use, and the dosing times of day on dental caries. They noticed that children who used medication more than twice daily experienced more caries in both primary and mixed dentition and also reported a temporal relationship between the medication time and an increase in incidence of caries in primary dentition. This particularly happened in children who had their dosing time before going to bed without cleaning or rinsing their mouth [45].

Two other studies outlined a higher percentage of caries in asthmatics with permanent dentition. In the first, caries were detected in 83% of patients with permanent dentition rather than those with primary (70%) or mixed (78%) ones [46]. The second study evidenced no increased risk of caries in primary teeth while the relative risk in permanent teeth was estimated at 1.45 (95% CI), especially in children taking both beta 2 agonists and inhaled steroids [47].

A cross sectional study conducted in 2016 on over 40 asthmatic patients between the ages of 18 and 65, evaluated the dental status using the DMFT index (decayed, missing, and filled teeth). Higher values of this score were found in asthmatics in comparison to controls, but the differences were not statistically significant (p = 0.199). For decayed and missing teeth suggesting a higher incidence of caries, the difference between the two groups was statistically different (p < 0.001). Extraction was, in fact, the most common therapy used for caries in the population studied, as the evaluated subjects had been suffering from the disease for about 5 to 10 years and they did not use any preventive dental care (partly due to the lack of information about the drugs’ side effects) [48].

It is important to outline that not only asthma per se and asthma treatment, but also concomitant rhinitis, can influence the incidence of caries. Rhinitis, as documented in ARIA (Allergic Rhinitis and its Impact on Asthma) guidelines, is present in almost 80% of asthmatic patients and could be a confounding factor in the pathogenesis of oral disease in asthmatics, and this has not been investigated in several studies [49].

A retrospective study conducted on 9038 children, observed for 9 years since birth, analyzed the correlation between asthma, rhinitis and caries and the influence of AR drugs in this condition. The authors concluded that there was not any correlation between dental caries and asthma and that the clinical visits for caries were increased by 13–25% in AR patients (p < 0.001). AR medications can also play a role in caries formation by decreasing the salivary flow rate. Second generation antihistamines generally have less anticholinergic effects than first generation ones, but in this study both drugs were used. Furthermore, topical nasal steroids can be connected, not only by reducing salivary flow, but also by changing oral flora. Even if not under specific treatment, AR patients could have increased levels of cariogenic bacteria and mouth breathing, which is a known risk factor for caries formation [50].

In conclusion, in order to reduce the incidence of dental cavities in asthmatics it is suggested to use sugar free medication and fluoridated products to rinse the mouth. Sugary medications and non-regular use of fluoridated rings positively correlate with an increased number of caries. Rinsing and gargling the oral cavity, using spacer devices, and trying to reduce the dosage and frequency of ICS are also recommended [44].

## Conclusion

Asthma and even more the therapeutic strategies that are needed in order to achieve the optimal disease control may significantly impact on oral health of affected patients. Patient related risk factors for oral diseases should be carefully assessed and compared with the anti-asthmatic drugs pharmacodynamics and pharmacokinetics characteristics that may amplify the patients’ predisposition. In this way asthma treatment selection and schedule can be tailored in order to minimize the impact of the drug on oral health. Also, when detecting oral diseases in asthmatic patients, a careful revision of both ongoing inhalation drugs and inhalation technique should be performed. Technical errors when assuming the drug may amplify the side effects in the oral districts, by increasing the deposition of drug particles on oral mucosa and dental surface. In fact, patient education plays a central role in maintaining oral health. In particular, oral hygiene, correct dietary habits, and an appropriate use of inhalers are essential.

Through a simple evaluation it is possible to optimize the treatment effectiveness and to practice a precision medicine approach for the global well-being of our patients.

## Data Availability

Data sharing not applicable to this article as no datasets were generated or analysed during the current study.

## Abbreviations

ICS: Inhaled corticosteroids
CSI: Corticosteroid inhalers
DPI: Dry powder inhalers
TH2: T Helper 2
FP: Fluticasone propionate
GERD: Gastroesophageal reflux disease
GOR: Gastroesophageal reflux
BEWE: Basic Erosive Wear Examination
GI: Gingival Index GI
OSA: Obstructive sleep apnea
LABA: Long-acting anticholinergic inhaled drug
